# A 0.85% saline as alternative detection buffer for SD-Bioline HIV rapid test in resource limited setting

**DOI:** 10.11604/pamj.2020.37.241.24234

**Published:** 2020-11-16

**Authors:** Shabani Iddi, Evangelista Malindisa, Musa Shija, Betrand Msemwa, Vitus Silago, Mariam Mirambo, Stephen Mshana

**Affiliations:** 1 Department of Physiology, Weill Bugando School of Medicine, Catholic University of Health and Allied Sciences, Mwanza, Tanzania,; 2 Department of Microbiology and Immunology, Weill Bugando School of Medicine, Catholic University of Health and Allied Sciences, Mwanza, Tanzania

**Keywords:** 0.85% saline, HIV infections, buffer, serologic tests

## Abstract

Accuracy in the diagnosis is a key step to identify HIV infected individuals for appropriate management. Insufficient supply of manufacturer´s buffers in relation to the number of strips per kit has negative impact on patient´s results hence improper patient´s management. In resource limited settings, some laboratory staff use different substitute buffers which has never been validated on their reliability. This study aimed at comparing the performance of 0.85% saline and SD-Bioline manufacturer´s buffer in detection of HIV antibodies. A total of 220 whole blood specimens: 110 HIV positive specimens from patients attending care and treatment center (CTC) and 110 HIV negative specimens from blood donors were re-tested for HIV status using SD-Bioline HIV rapid test using manufacturer´s buffer and 0.85% saline separately. Data and laboratory results were recorded in Microsoft excel sheet followed by analysis using STATA version 13. For all tested samples, the level of agreement between 0.85% saline and manufacturer´s buffer was 98.64% (kappa=0.9727). The value of kappa indicates very good agreement between 0.85% saline and manufacturer´s buffer. In incidents where manufacturer´s buffer is not sufficient, 0.85% saline can give reliable results. Further studies to evaluate the suitable buffer for other rapid tests for HIV and other diseases are recommended especially in resource limited settings.

## Introduction

Human Immunodeficiency Virus/Acquired Immunodeficiency Syndrome (HIV/AIDS) is the pandemic disease that is one of the most serious global socio-economic and health problem facing people of all ages in the world [1]. HIV/AIDS have brought negative impacts to the communities as well as workplaces. The major impacts include reduction of labor supply and increased medical expenditure hence reduction of productivity in all sectors. Due to this global disaster, there have been establishment of different strategies aimed at HIV prevention and control in the community. World Health Organization (WHO) has introduced programs for promoting and protecting the community against HIV/AIDS including providing access to information and education to the countries worldwide as well as instituting management and control strategies such as early detection of HIV infection and provision of appropriate care. Early and accurate detection of HIV infection is important in order to identify infected individuals. There has been advancement of diagnostic methods following a call for universal access to prevention, care and treatment by the WHO with introduction of number of point of care tests [2]. Therefore, there is a need to ensure that all available and mostly used diagnostic methods provide a reliable result which can be useful and important part of reaching a goal of reducing consequences of HIV/AIDS pandemic.

Following expansion of market for HIV diagnostics in recent years, there are a number of rapid test kits which are recommended as per different countries diagnosis algorithms. Nevertheless, not all kits are often manufactured with acceptable standards, some have been supplied with insufficient buffers. SD-Bioline® HIV diagnostic test kits are usually packed with a single diluent buffer whereby in some occasions the buffer supplied tends to be insufficient for all cassettes supplied. In resource limited settings some laboratories personnel uses different substitute buffers including normal saline, tap water and diluents from other manufacturers such as Unigold and malaria rapid diagnostic test (MRDT). The substitution of the kits buffers has been proved to give a good number of false positive and false negative results. Studies have been done on comparing substitute buffers with commercially provided buffers in some HIV Rapid Test Kits (RTKs) but none have been done on SD-Bioline [3-5]. This study has provided information on the usefulness of 0.85% saline buffer as detection buffer for SD-Bioline® HIV test, and therefore provide suggestive alternatives to be used as substitute buffers in resource limited settings.

## Methods

This laboratory-based study was conducted between May and July 2018 at Microbiology laboratory of the Catholic University of Health and Allied Sciences (CUHAS) in Mwanza, Tanzania. A total of 220 whole blood samples were collected whereby 110 were from known HIV positive patients attending care and treatment center (CTC) of the Bugando Medical Centre and 110 were from known HIV negative blood donors from the Lake zone blood bank. All HIV positive specimens were screened and documented their results based on Tanzania rapid test algorithm [6] while all HIV negative were confirmed by 4^th^ generation Enzyme Linked Immunosorbent Assay (ELISA) (Bioelisa HIV1/2 ver.4.0-Spain). All samples were supplied without any identifier to client apart of serial labelling and no socio-demographic and clinical information were provided. The samples were aliquoted to produce two groups (A and B) each with 220 samples and all were tested using SD-Bioline kit. Group A sample were tested with SD-Bioline using manufacturer´s buffer (Standard Diagnostic Inc 65, Borahagal-ro, Giheung-gu, Yongin-si, Gyeonggi-do, Republic of Korea) and group B samples with SD-Bioline using 0.85% saline as buffer (Loba Chemie Pvt Ltd 107, Wodehouse road, Mumbai, India). The RTKs used for this study were of the same Lot number and expiry date and were used within their expiry date. The refrigerated samples were pre-warmed at room temperature before testing. The RTKs were subjected to internal quality control testing with both positive and negative specimen before running with the participants´ blood using manufacturers´ buffer and the substitute buffer (0.85% saline). All tests were performed according to manufacturer´s instruction. Readings were performed at daylight after the prescribed 10 to 20 minutes incubation period after application of blood sample and buffer or blood sample and 0.85% saline. RTK test lines were interpreted according to the manufacturer´s instructions.

Data were recorded in Microsoft excel sheet followed by analysis using STATA version 13 and Kappa statistics was used to calculate agreement and disagreement between 0.85% saline and SD-Bioline manufacturer´s buffer. This laboratory-based study was approved by the joint Catholic University of Health and Allied Sciences/Bugando Medical Centre Research Ethics and Review Committee (CREC) with ethical clearance certificate number CREC/603/2018.

## Results

Of the total specimens tested, 107 (97.3%) of HIV positive were positive by using both SD-Bioline buffer and 0.85% saline buffers while 110 (100%) of HIV negative samples were negative by both SD-Bioline buffer and 0.85% saline. The value of kappa indicates very good agreement between 0.85% saline and SD-Bioline manufacturer´s buffer (Table 1).

**Table 1:** distribution of HIV results by SD-Bioline® HIV rapid test using manufacturer's buffer and 0.85% saline

Normal saline	Chase buffer positive 110	Chase buffer negative 110	Total	Kappa
**Positive**	107 (97.3%)	0 (0%)	107	
**Negative**	3 (2.7%)	110 (100%)	113	
**Total**	110	110	220	0.9727

## Discussion

Saline 0.85% has been studied as detection buffer for rapid test and has recorded a sensitivity and specificity of 100% [3,4]. We hereby for the first time document the usefulness of 0.85% saline as a detection buffer for HIV SD-Bioline rapid test. The good agreement obtained in this study is due to the fact that 0.85% saline is used for human and animal therapeutic purpose as well as in different biological research in clinical settings. Similar results were obtained in the study done in Nigeria about the use of normal saline as alternative buffer in Alere determine for HIV test [4]. The finding of this study may provide alternative means of diagnosing HIV with maintained quality of results in case of shortage of buffer that can lead to proper management of patients especially in resource limited settings instead of re-ordering of new kits that may be associated with increased running costs.

## Conclusion

The 0.85% saline can provide same reliable results as the SD-Bioline manufacturer´s buffer. While manufacturer´s buffer is always preferred, we recommend that 0.85% saline can be used in cases where SD-Bioline manufacturer´s buffer is insufficient for SD-Bioline HIV rapid test for whole blood samples. This will ensure that HIV testing for those seeking the test is timely done. More studies to evaluate suitable alternative buffers for other rapid tests for different diseases are recommended especially in resource limited setting.

### What is known about this topic

- Phosphate buffered saline gives same reliable results as the Alere determine buffer supplied by manufacturers in HIV testing;
- Sterile normal saline testing with determine does not give false positive or false negative test results in HIV testing.

### What this study adds

- Percentage agreement between SD-Bioline manufacturer´s buffer and 0.85% saline in SD-Bioline rapid test for HIV;
- Usefulness of 0.85% saline as alternative detection buffer for SD-Bioline HIV rapid test.
